# Biosafety conducts adopted by orthodontists

**DOI:** 10.1590/2177-6709.23.3.073-079.oar

**Published:** 2018

**Authors:** Camila Gonçalves Jezini Monteiro, Mariana Martins e Martins, Adriana de Alcantara Cury-Saramago, Henry Pinheiro Teixeira

**Affiliations:** 1 Universidade Federal Fluminense, Faculdade de Odontologia (Niterói/RJ, Brazil).; 2 Universidade Federal Fluminense, Departamento de Ortodontia (Niterói/RJ, Brazil).; 3 Odontoclínica Central da Polícia Militar (Rio de Janeiro/ RJ, Brazil).

**Keywords:** Sterilization, Disinfection, Orthodontics, Personal protective equipment

## Abstract

**Objective:**

This cross-sectional observational study was designed to assess the biosafety conducts adopted by orthodontists, and possible differences regarding training time.

**Methods:**

Both the application of methods for sterilization/disinfection of instruments and materials, and the use of personal protective equipment (PPE) were collected through questionnaires via e-mail.

**Results:**

The questionnaires were answered by 90 orthodontists with a mean age of 37.19 ± 9.08 years and mean training time of 13.52 ± 6.84 years. Regarding orthodontic pliers, 63.23% use an autoclave, except 1 who does not perform any procedure. All participants use autoclave to sterilize instruments, and 95.6% of respondents perform cleaning with chemicals prior to sterilization. Most of them (65.56%) use an autoclave to sterilize orthodontic bands, with some still associating disinfection methods, while few (18.89%) do nothing at all. There was a high incidence of the answer “nothing” for the methods used for elastic, accessories, bandages, metal springs, and arches. All respondents use mask and gloves in attendance, 78.92% use aprons, 58.92% use protective goggles, and 50.01% use cap. Training time significantly influenced (*p* = 0.003) only the use of glutaraldehyde for sterilization/disinfection of pliers.

**Conclusions:**

The sterilization and cleaning of pliers, instruments, and bands, besides the use of PPE, received more uniform and positive responses, while other items suggest disagreements and possible failures. Only orthodontists trained for more than 13 years choose using glutaraldehyde for pliers sterilization/disinfection, the only adopted method with a significant difference in relation to training time.

## RESUMO

**Objetivo:**

o presente estudo observacional transversal foi delineado para avaliar as condutas de biossegurança adotadas pelos ortodontistas, e as possíveis diferenças em função do tempo de formação.

**Métodos:**

tanto as técnicas de esterilização e desinfecção de instrumentais e materiais quanto o uso de equipamentos de proteção individual da equipe foram coletados por meio de questionários via e-mail.

**Resultados:**

os questionários foram respondidos por 90 ortodontistas, com idade média de 37,19 ± 9,08 anos e tempo médio de formação de 13,52 ± 6,84 anos. Quanto aos alicates ortodônticos, 63,23% usam autoclave, exceto aqueles que não realizam nenhum procedimento. Houve total abrangência no uso da esterilização do instrumental por autoclave, sendo que 95,6% realizam a limpeza com substâncias químicas previamente à esterilização. A maioria (65,56%) utiliza autoclave para a esterilização de anéis ortodônticos e alguns associam métodos de desinfecção, mas 18,89% nada fazem. Houve alta incidência da resposta “nada” para os métodos utilizados em elásticos, acessórios, ligaduras, molas e arcos. Todos os questionados utilizam máscara e luva no atendimento ortodôntico, 78,92% incluem o uso do avental, 58,92% usam óculos de proteção e 50,01% vestem o gorro (50,01%). O tempo de formação influenciou significativamente (*p*= 0,003) apenas no uso do glutaraldeído para esterilização e/ou desinfecção dos alicates.

**Conclusões:**

a esterilização e limpeza de alicates, instrumental de exame, aplicador de elásticos e anéis ortodônticos, além do uso de equipamentos de proteção individual, receberam respostas mais uniformes e positivas, enquanto os outros itens sugerem falhas. Apenas os ortodontistas formados há mais de treze anos optaram pelo uso do glutaraldeído para esterilização/desinfecção dos alicates ortodônticos, sendo a única conduta que mostrou diferença significativa em relação ao tempo de formação.

## INTRODUCTION

The concept of biosafety was introduced in the 1970s at the Asilomar meeting in California where the scientific community began discussing the impacts of genetic engineering on society. This meeting, according to Albuquerque,^1^ was a milestone in the history of ethics applied to research, representing the first time in which aspects of protection to researchers and other professionals involved in areas in which research projects are carried out were discussed. Since then, the term biosafety has been changing over the years, and is currently considered to be the set of actions aimed at prevention, minimization or elimination of risks inherent to activities and research, production, teaching, development, technology, and service provision aimed at the health of man and animals, the preservation of the environment and the quality of results.^2^

The oral cavity is the site of the greatest concentration of microorganisms inside the clinic, making the clinical environment conducive to exposure to biological risks.^3^

Research shows that a universe of pathogenic microorganisms is hidden in dentistry instruments^4^ including, among others, hepatitis B, HIV, hepatitis delta, herpes, and influenza, besides the tuberculosis bacillus.^5^ ^,^ ^6^

Orthodontic clinical routine is characterized by several possible disease-transmitting vehicles and by the high turnover of patients, which implies an increasing frequency of handling the material and, consequently, the need to maintain the equipment and the entire care structure free of pathogenic microorganisms.^4^ ^-^ ^8^

Besides all care taken with the articles used during orthodontic treatment against possible biological risks, the professional and his/her team, as well as the patient, should be protected, each with the appropriate personal protective equipment (PPE), including gloves, mask, coat, goggles, and cap.^2^ ^,^ ^9^

In Brazil, there is no defined biosafety conduct protocol, but there are norms and guidelines of ANVISA (National Sanitary Surveillance Agency) that help the orthodontist in his routine. According to ANVISA, most of materials and instruments used in orthodontics are classified as semi-critical, because they come into contact with mucosal or non-intact skin, requiring sterilization or high-level disinfection.^2^ In this way, autoclaving should be the first choice, for being the only form of sterilization. However, there are some materials that may suffer mechanical properties damage when autoclaved.

The literature on the subject individually addresses the sterilization or disinfection of specific items, such as pliers,^4^ ^,^ ^10^ ^-^ ^15^ elastics,^16^ ^-^ ^22^ and orthodontic bands,^23^ ^-^ ^26^ among other items.^27^ In addition, there are few reported questionnaires, which work as an important tool to verify if the most appropriate methods are actually used by orthodontists. Among the few questionnaires found in the area of orthodontics, two evaluate the sterilization behavior of only one specific item, such as orthodontic bands^26^ and photograph retractors;^27^ and only one more broadly evaluates the conducts regarding the sterilization and disinfection procedures adopted in the orthodontic clinic^23^ - a questionnaire carried out more than 5 years ago in the Northern region of Brazil.

Thus, the need to better know the profile of the conduct adopted by orthodontists in relation to various instruments, orthodontic materials, and the use of PPE, not just certain items, is evident. Therefore, the present study evaluated the biosafety conducts adopted in orthodontic care in the state of Rio de Janeiro (Brazil), regarding the techniques used to sterilize and disinfect some instruments and materials, and regarding the use of PPE by orthodontists; and also sought to evaluate possible differences between responses depending on training time.

## MATERIAL AND METHODS

This research was classified as a cross-sectional observational study, and was approved by the Research Ethics Committee of the *Universidade Federal Fluminense* (protocol #1,430,226).

The answers obtained from the questionnaire were used as material for this research. A sample calculation was performed based on the data provided by the Regional Council of Dentistry of the state of Rio de Janeiro (RCD-RJ), which indicated the presence of 1,496 registered orthodontists.

The formula below for small populations^28^ was applied in which Zα was set at 1.96, since the confidence level adopted was 95%; *p* was set at 0.5, to act more conservatively and have the largest possible size of the sample; N was the population number (1,496) and Cp was the confidence interval, set at 10% (0.10), resulting in the need for 90 participants for the survey.

$$n=\frac{Z\alpha^{2}[p(1-p)N}{Z\alpha^{2}[p(1-p)]+(N-1)Cp^{2}}$$

The questionnaire was composed of one question with eight subitems to indicate the biosafety technique used, two questions on the disinfection of instruments, and one on PPE. The orthodontists were informed that they could respond with more than one option for the same question and that they had the option of identifying themselves or not.

Orthodontists were invited to participate in the study via e-mail. A list of electronic addresses was created by selecting all the orthodontists in the state of Rio de Janeiro using internet. Were selected all those who had their education or worked in that state. E-mails were sent to everyone on the list and the first 90 orthodontists who responded and met the inclusion criteria were selected to participate in the survey.

The inclusion criteria consisted of specialists trained in specialization courses recognized by the Ministry of Education and Culture (MEC) and who trained or work professionally in the state of Rio de Janeiro. Orthodontists who did not correctly complete the questionnaire and/or did not send the signed informed consent form were eliminated.

The BioEstat 5.3 software (Belém/PA, Brazil) was used to obtain the mean, standard deviation, maximum and minimum values of the age and training time of the orthodontists, and to analyze possible differences in conduct according to the training time, applying the G-test. A distribution analysis of the questionnaire responses was also performed through the percentages of answers for each question.

## RESULTS

The sample consisted of 90 specialists in orthodontics, 53 (58.8%) female and 37 (41.1%) male. As for age, the mean was 37.19 ± 9.08 years, maximum 65, and minimum 25 years. Regarding training time, the mean was 13.52 ± 6.84 years; the longest training time was 32 years and the shortest was 4 years.

The results for the eight subitems of the first question of the questionnaire are shown in Table 1. Open-answers were used to describe the subitems “other methods” and “associations”.

**Table 1:** How and what is the method of sterilization and/or disinfection that you use for orthodontic care?

METHODS	Pliers		Elastics		Accessories		Metal ligatures		Instruments		Bands		Springs		Arches	
	n	%	n	%	n	%	n	%	n	%	n	%	n	%	n	%
Autoclave	30	33.33	2	2.22	15	16.67	14	15.56	86	95.56	59	65.56	10	11.11	9	10
70% alcohol	24	26.67	38	42.22	9	10	11	12.22	0	0	7	7.78	12	13.33	23	25.56
Oven	0	0	0	0	1	1.11	0	0	0	0	0	0	0	0	0	0
Glutaraldehyde	0	0	3	3.33	2	2.22	1	1.11	0	0	1	1.11	2	2.22	1	1.11
Peracetic acid	2	2.22	3	3.33	1	1.11	3	3.33	0	0	2	2.22	3	3.33	2	2.22
Nothing	1	1.11	36	40	60	66.67	58	64.44	0	0	17	18.89	58	64.44	50	55.56
Other methods	2	2.22	4	4.44	0	0	2	2.22	0	0	0	0	2	2.22	2	2.22
Associations	31	34.44	4	4.44	2	2.22	1	1.11	4	4.44	4	4.44	3	3.33	3	3.33

In the subitem “pliers”, those who answered “other methods” cited a sterilizer oven at 350^o^C and chlorhexidine wipes, as well as washing with water and detergent, and an ultrasonic tub. There were 11 associations of different methods of sterilization and/or disinfection of pliers. Among them, the most frequent was 70% alcohol with sterilization in an autoclave (21%).

In relation to elastics, two associations were found: 70% alcohol and glutaraldehyde (3.3%), and 70% alcohol and an ultrasonic tub with enzymatic detergent (1.1%).

Responses related to accessories also had two associations: 70% alcohol and glutaraldehyde (1.1%), and autoclave and sterilizer oven (1.1%). The other method reported for sterilization/disinfection of metal ligatures was formalin tablets, and only one combination was obtained: 70% alcohol, oven, and autoclave (1.1%).

For instruments (tweezers, probe, mirror, and ligature adapter), there were three different associations: 70% alcohol and autoclave (2.2%); oven and autoclave (1.1%); water and soap, ultrasonic tub, and autoclave (1.1%).

Regarding the bands, the data showed three associated methods: 70% alcohol and autoclave (2.2%); oven and autoclave (1.1%); water and soap, ultrasonic tub, and autoclave (1.1%).

For springs, two associations were reported: 70% alcohol and glutaraldehyde (2.2%), and oven and autoclave (1.1%). Regarding arches, the other methods pointed out were washing with soap and water; 70% alcohol and lamp; and an autoclave when used in the same patient. Autoclave and oven (1.1%), and 70% alcohol and glutaraldehyde (2.2%) associations were found.

Table 2 expresses the results of the second question. In this item, in “other methods” were reported: Washing with soap and water, ultrasonic tub, enzymatic detergent, brushing, and biocide solutions. The most used association was washing pliers with soap and water and 70% alcohol (38.46%).

**Table 2:** How and what is the method of cleaning pliers and instrumentals that you use before sterilization and/or disinfection?

Methods	N	%
Glutaraldehyde	7	7.78
NaOCl 1%	2	2.22
70% alcohol	28	31.11
Nothing	4	4.44
Chlorhexidine 0,12%	4	4.44
Others	32	35.56
Associations	13	14.44

The results of the third question are shown in Table 3. The PPE listed were: Gloves, mask, cap, goggles, and apron. Data were divided by the number of associated PPE items.

**Table 3:** Use of personal protective equipment (PPE) by the orthodontist.

PPE	N	%
A, B, C, D, E	26	28.89
A, B, C, E	4	4.44
A, B, D, E	19	21.11
A, C, D, E	13	14.44
A, B, E	4	4.44
A, C, E	1	1.11
A, D, E	13	14.44
A, E	9	10
Sem resposta	1	1.11

A) gloves; B) goggles; C) cap; D) apron; E) mask.

The sample was subdivided, regarding the mean training time (13.52 ± 6.84 years), between participants with up to 13 years of training and participants with more than 13 years of training, to evaluate possible differences in conducts; a difference was found (*p*= 0.003) only in the use of glutaraldehyde for sterilization and/or disinfection of pliers, which was only done by professionals with a longer training time (Table 4). Some evaluations could not be performed due to the absence of yes or no responses in the two groups.

**Table 4:** G-test to assess possible differences in conduct between participants with up to 13 years of training and participants with more than 13 years of training.

Sterilization/Disinfection (TAB 1)	Pliers	Elastic	Accessories	Metal Ligatures	Instruments	Bands	Springs	Arches
Autoclave	0.7065	0.6073	0.8866	0.2147	X	0.7547	0.816	0.6209
70% alcohol	0.9269	0.0252	0.8507	0.7856	0.8229	0.2297	0.3495	0.8185
Oven	0.6073	X	0.6073	0.8749	0.1872	0.8749	0.8749	0.8749
Glutaraldehyde	0.003*	0.4117	0.7827	0.8749	0.1872	0.8749	0.0535	0.1355
Peracetic acid	0.7187	0.1355	0.8749	0.1355	X	0.3317	0.3412	0.6073
Nothing	0.8749	0.24	0.5974	0.1142	X	0.9574	0.0753	0.844

G) Other methods? Which? ___________.

## DISCUSSION

The sample was homogeneous in relation to gender distribution, but heterogeneous regarding age and training time of the interviewees. The age ranged from 25 to 65 years and the training time between 4 and 32 years. This large variation significantly influenced only the use of glutaraldehyde for sterilization and/or disinfection of pliers, which was only done by professionals with longer training periods.

Orthodontic pliers can be used only in the laboratory or during clinical care, when they come into direct contact with patients’ mouths, being potential transmitters of microorganisms.^10^ This instrument can be classified as a semi-critical article when used in clinical care because it comes into contact with non-whole skin or mucous and, therefore, requires a high level sterilization or disinfection.^2^ ^,^ ^11^ ^-^ ^14^ ^,^ ^29^

However, some orthodontists believe in disinfection as an alternative to sterilization, which is consistent with the results of the present study, in which the method of sterilization and/or disinfection of choice was the use of 70% alcohol, which promotes disinfection only.^4^

Several studies show that it is common to wash orthodontic pliers with soap and water, followed by disinfection with 70% alcohol, a questionable and insufficient method for disease control.^10^ In addition, residual bacteria are present in large quantity and variety after this procedure.^11^ The study by Carvalho et al.^12^ showed that only 0.025% peracetic acid and 2% glutaraldehyde are able to disinfect orthodontic pliers, and that 70% alcohol is ineffective to eliminate *Staphylococcus aureus*. Ratifying the inefficiency of 70% alcohol, Almeida et al.^15^ showed that, using this method, 20% of pliers remain infected and that immersion of the pliers in 2% glutaraldehyde is able to decontaminate all orthodontic pliers. However, this conduct is inappropriate due to the cytotoxic characteristics of glutaraldehyde.^2^ ^,^ ^30^

Of those interviewed, 63.23% use an autoclave as a means of sterilization, with 33.33% of them using an autoclave only, while 29.90% associate autoclaving with another sterilization/disinfection method. Only one person does not perform any method (Table 1). Badaró et al^23^ showed that 68.75% use the autoclave as their first choice, similar to this research; 18.75% disinfect only with 70% alcohol, another 12.5% report the use of glutaraldehyde, and 6.25% dry heat oven. The results of the two surveys show that more than half of the interviewees use an autoclave, an adequate method of sterilizing orthodontic pliers.^23^ However, further studies and publicity and surveillance campaigns are needed by the competent bodies to encourage the adoption of effective and up-to-date biosafety standards by all orthodontists.

All the orthodontists questioned use autoclave sterilization of instruments (probe, tweezers, mirror, and ligature adapter) (Table 1). Most clean them with chemical substances prior to sterilization and only 4.4% do not do this (Table 2). These data are very positive and indicate the presence of an autoclave in the clinics, suggesting that not using it for the pliers could be due to the fear of damaging them, as they are considered an expensive item.^16^

However, manufacturers’ instructions exist to minimize the effects of corrosion that may occur with the use of an autoclave. Some authors emphasize that corrosion is linked to the medium used and to the metal composition of each pair of pliers, and disinfection with chemical solutions is possibly more damaging.^13^ ^,^ ^14^

Most of the interviewees (65.56%) use an autoclave for the sterilization of orthodontic bands. In addition, some use associated disinfection methods. Unfortunately, 18.89% do nothing for the biosafety of this item. Fulford, Ireland, and Main^24^ found that the use of enzymatic detergents together with subsequent autoclaving of orthodontic bands eliminates all forms of bacteria, making it safe to reuse tested and unused bands. Badaró et al^23^ found that 87.5% of those interviewed autoclaved before returning them to the case; 31.25% wash them under running water and store them; another 31.25% immerse bands in chemical solutions for more than 10 hours; and 12.5% do nothing.

The questionnaire of the present study did not specify whether or not the bands were tried. The lack of this information probably represents a limitation of the study.

The high incidence of the “nothing” response for the methods used for sterilization and disinfection of elastics, accessories, metal bandages, springs, and arches (Table 1) was not surprising, especially since there is not enough research on all these materials.^16^ ^-^ ^19^ Regarding elastics, Mayberry et al^20^ found that there was degradation (shrinkage) after autoclaving with a 20 minute cycle. Evangelista, Berzins, and Monaghan ^19^ exposed elastics to a disinfectant solution for an hour or more, by which time the strength decreased. Pithon et al^21^ did not find any changes in the mechanical properties of elastics after undergoing different forms of sterilization. The majority of studies evaluated the use of glutaraldehyde solutions that are no longer used today due to the associated cytotoxic effects.^30^ There are few publications related to peracetic acid, which has replaced glutaraldehyde for disinfection of these and other orthodontic materials.^21,22^ Mattos^22^ states that, despite being non-toxic and effective against microorganisms, peracetic acid has a negative effect on elastic properties, increasing plastic deformation, and concluding that the ideal would be an autoclaving cycle as prior sterilization of elastics. Pithon et al^21^ also evaluated peracetic acid. However, as previously mentioned, they did not find changes in the mechanical properties of the elastics. Thus, the lack of consensus in the literature and the need for further research on the subject is evident.

For the method of sterilization/disinfection of elastics, 42.22% of respondents stated that they use 70% alcohol, while 40% do nothing. Chemical solutions described in the questionnaire and other methods were pointed out, with minor incidence (Table 1). The proportion of those who answered “nothing” for the sterilization of elastics is similar to that in the study by Badaró et al.^23^

The behavior of those interviewed was similar for the biosafety conducts regarding metal ligatures, springs and orthodontic arches. Most of these orthodontists do not perform sterilization and disinfection procedures for these items, while some use 70% alcohol; practically the same values were obtained for those using an autoclave, peracetic acid, or glutaraldehyde.

Glutaraldehyde solutions have a cytotoxic effect on the skin and mucous membranes, and have a reduced shelf-life when diluted.^2^ In light of these facts, the responses that indicated the use of glutaraldehyde were considered outdated and, fortunately, only a small portion of those interviewed used this method. It was possible to verify that only orthodontists with more than 13 years of training used glutaraldehyde for sterilization/disinfection of orthodontic pliers, the only conduct that showed a significant difference in relation to training time.

PPE should be used to protect the patient and the team involved in care.^5^ ^,^ ^9^ All respondents use a mask and gloves in orthodontic care. Most of the answers included the use of an apron and goggles. As for a cap, the answers were equally divided (Table 3). Despite this, the results are positive, since 68.84% of the sample uses a set of four to five associated PPE items. Likewise, Pereira^9^ found that goggles have the second lowest incidence of use among these professionals (57.1%), preceded by the use of a cap (13.3%), when applying a questionnaire to 203 orthodontists in order to evaluate the methods of infection control in the orthodontic offices of the city of Rio de Janeiro.

Some data obtained by this research are alarming and worrying, as more than 60% of orthodontists didn’t use disinfection methods to metal ligatures, springs, accessories, as well 40% to elastics and 18,89% to bands. 

Awareness-raising policies should be encouraged among orthodontists, considering that the oral cavity is an environment rich in microorganisms and therefore of biological risk^3^; and that the high turnover of patients in the orthodontic clinic demands greater control of the cross infections.^4^ ^-^ ^8^

However, there are few literature studies on the best course of action to be adopted for these items,^16^ ^-^ ^19^ but there is a clear need for research aimed at the best form of sterilization or disinfection for these materials, since it is unacceptable that no procedure is done.

There is no validated questionnaire within the topic of applied biosafety in Orthodontics. Other studies with questionnaires^23^ ^,^ ^26^ ^,^ ^27^ also did not use validated instruments. In this way, it was not feasible to test the convergent and divergent construct validity of the instruments through hypotheses, to test correlations with other instruments. And, since a retest was not made, it was impossible to evaluate the reliability of the answers, being considered limitations of our study. It is suggested that new studies should be carry out to create a validated questionnaire for use in research on this topic.

Study of biosafety in Orthodontics is a challenging subject due to the large number of bacteria being tested and various types of material used in clinical care. Each item should be tested for all microorganisms according to the type of sterilization or disinfection that the material allows, without its physical and mechanical properties being impaired.

Biosafety applied to human health and, specifically, to dentistry, is much more comprehensive than the one issue addressed here. Other subjects are the object of studies, such as waste disposal, preparation of the work environment, preparation of plaster models, immunization of the involved professionals, disinfection of patients, accidents at work, and occupational risks.

Publications are expected to be related to the most diverse biosafety issues to raise awareness that biosafety is an extremely important factor in clinical routine.

## CONCLUSIONS

Orthodontists are concerned about applying biosafety control standards. However, a variety of behaviors was verified for the questioned items. The sterilization and cleaning of pliers, instruments, and orthodontic bands, and the use of PPE were the items that received the most uniform and positive responses, while responses to the other items suggest failures.

Only orthodontists trained for more than 13 years opted for the use of glutaraldehyde for sterilization/disinfection of pliers, the only conduct with a significant difference in relation to the training time.

The diversity of procedures that have been reported by these orthodontists suggests that more research is needed to provide guidance on the most effective method of decontamination and awareness-raising policies should be encouraged among these professionals.
